# Perspective: mitochondrial STAT3 in cardioprotection

**DOI:** 10.1007/s00395-023-01003-3

**Published:** 2023-08-24

**Authors:** Petra Kleinbongard

**Affiliations:** Institute for Pathophysiology, West German Heart and Vascular Center, University of Essen Medical School, Hufelandstr. 55, 45122 Essen, Germany

**Keywords:** Cardioprotection, Ischemia/reperfusion injury, Mitochondria, SAFE pathway, STAT

## Abstract

Activation of signal transducer and activator of transcription 3 (STAT3) has been identified as a key cardioprotective signal not only in animal studies but also in humans—in animals, STAT3 is causally involved in cardioprotection. In response to late ischemic conditioning, canonical function of STAT3 activation upregulates the expression of cardioprotective and anti-apoptotic proteins. In its non-canonical function, STAT3 is activated during ischemic conditioning and is part of the cardioprotective cytosolic survival activating factor enhancement pathway. Activated STAT3 is imported and localized to the mitochondria. Mitochondrial STAT3 stimulates the activity of mitochondrial electron transport chain complex I, reduces mitochondrial reactive oxygen species production and mitochondrial permeability transition pore opening. Finally, two novel aspects of STAT activation in cardioprotection are discussed: a genetic variance of the STAT encoding region as a potential primordial confounding variable for cardioprotection, and the cardioprotective potential of sodium–glucose cotransporter 2 inhibitors through STAT3 activation.

## Cardioprotection: strategies and relevance of STAT signaling

In addition to rapid reperfusion and current therapy there is still a need for cardioprotection to reduce morbidity and mortality in patients with acute myocardial infarction. In experimental settings, there are mechanical and pharmacological interventions that reduce myocardial infarct size. The strongest and most robust cardioprotective intervention is ischemic conditioning, which is effective in all species tested so far, including humans [45, 52]. Reduction of infarct size by ischemic conditioning can be induced by cycles of brief ischemia/reperfusion before (ischemic preconditioning, IPC) [79] or after (ischemic postconditioning, POCO) [119] sustained myocardial ischemia with reperfusion. Ischemic conditioning can also be induced remotely from the heart (remote IPC, RIC) [43, 45]. Among these cardioprotective strategies, RIC has been successfully translated from experimental studies to clinical trials. In patients undergoing elective surgical coronary revascularization, there are several single-center trials in which RIC provided perioperative myocardial protection (e.g., [15, 40, 57, 89, 100, 103]), and one of them also reported improved patient prognosis [65, 100]. However, two prospectively designed multi-center phase III trials in patients undergoing elective surgical coronary revascularization and valve surgery, i.e., ERICCA and RIPHEART, were neutral [37, 77], possibly because use of propofol rather than volatile anesthesia [49]. Similarly, in patients with acute myocardial infarction, RIC attenuated myocardial injury in single-center trials (e.g., [13, 23, 108, 115]), and again one of them also reported an improved patient prognosis [94]. However, the prospectively designed larger phase III multi-center follow-up CONDI-2/ERIC-PPCI trial was neutral on myocardial injury and clinical outcome [39]. Only the prospectively designed single-center RIC-STEMI trial truly reported an improved clinical outcome as a primary endpoint with RIC [29]. A detailed and more comprehensive review of available clinical trials on cardioprotective strategies is found in: [42, 45]. Potentially confounding factors of the cardioprotective strategies in patients [24, 61, 90] as well as errors in the planning and design of preclinical and clinical trials are discussed in detail in the other reviews [44, 47, 70]. Irrespective of all these valid considerations, one reason for the lack of success in translating cardioprotective strategies from experimental studies to the clinical situation is that the underlying signaling pathways are incompletely understood and much more basic research is needed to improve our understanding of the signaling pathways involved in cardioprotective interventions that are in principle applicable.

**Fig. 1:**
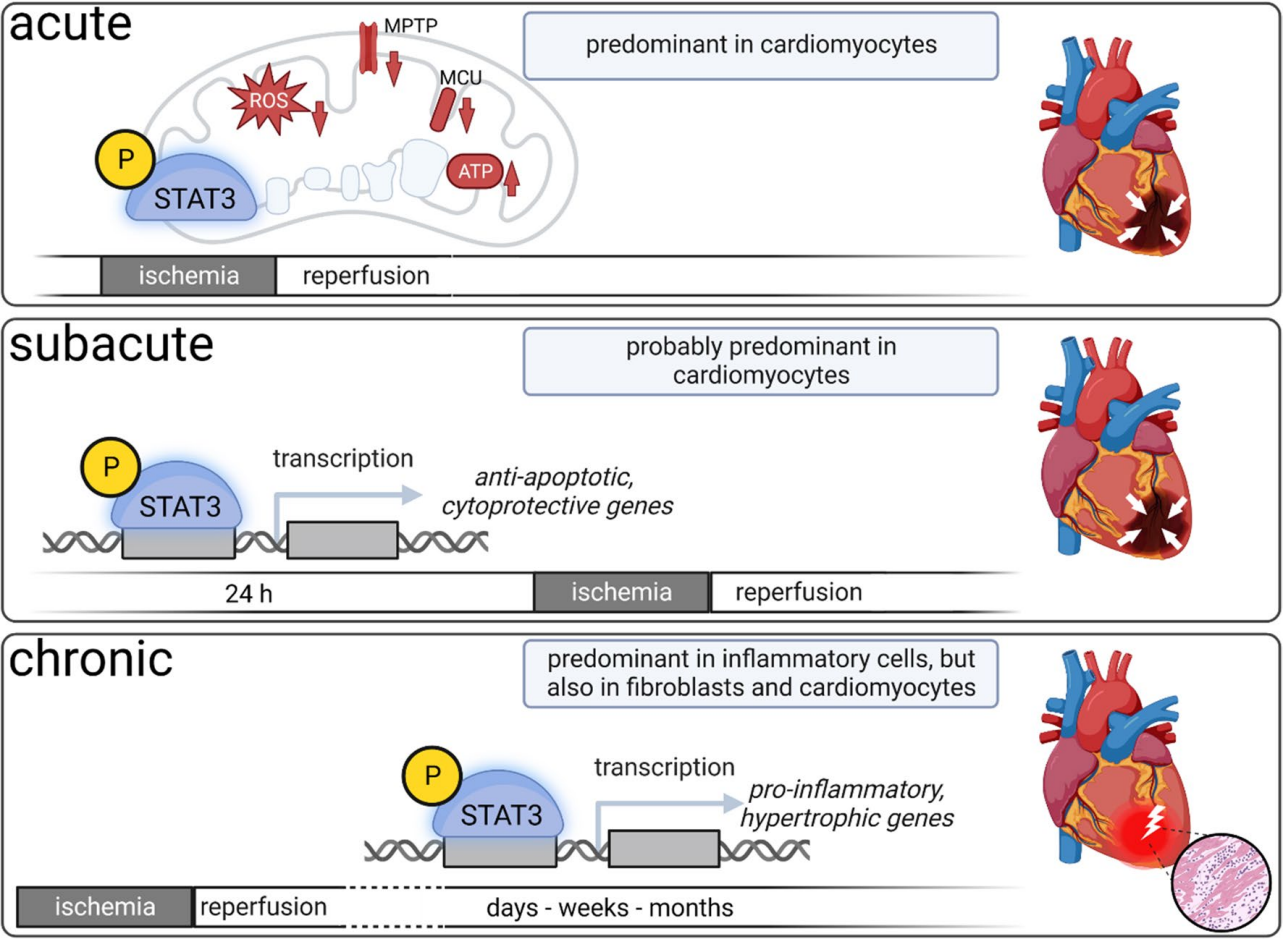
Schematic overview of the time-dependent—acute, subacute, chronic—and non-canonical vs. canonical effects of STAT3 activation on myocardial ischemia/reperfusion; created with BioRender.com. *ATP* adenosine triphosphate, *MPTP* mitochondrial permeability transition pore, *ROS* reactive oxygen species, *STAT3* signal transducer and activator of transcription 3

Currently, the underlying myocardial signal transduction of cardioprotection [43, 45] can be conceptually classified by their (sub-) cellular localization (extracellular molecules, cytosolic signal transduction and target organelle/structure). Extracellular molecules (e.g., autacoids, calcium, cytokines, neurohormones, nitric oxide, or reactive oxygen species) are released during conditioning cycles from cardiomyocytes, endothelial cells, neurons, etc., but the exact subcellular origin and detailed biochemical reactions of how these extracellular molecules are generated and released are unclear. Through sarcolemmal receptors or receptor-independently, these molecules then activate cytosolic signaling cascades. Within the cardiomyocyte, a variety of proteins are activated as cytosolic signal transducers. Again, conceptually, cytosolic signaling pathways are divided into three major cardioprotective pathways: the nitric oxide/protein kinase G (NO/PKG) pathway [20], the reperfusion injury salvage kinase (RISK) pathway [41], and the survival activating factor enhancement (SAFE) pathway [69]. The RISK pathway and its interaction with mitochondrial function is the subject of a detailed discussion in the current issue of “Mitochondria at the heart of cardioprotection” [116]. Key protein of the SAFE pathway is the signal transducer and activator of transcription (STAT)3 [6, 43, 45, 67, 69]. In response to ligand binding [e.g., interleukin 6-like cytokines, tumor necrosis factor alpha (TNF)] through sarcolemmal glycoprotein 130 or TNF receptors, Janus kinase (JAK) is activated and phosphorylates STAT3 [tyrosine (tyr) 701 and serine (ser) 727]. STAT3 phosphorylation is required for the protein dimerization, its subsequent translocation to the nucleus, and its function as a transcription factor; the ser727 phosphorylation seems to be the boost for transcriptional activity of STAT3 [26]. STAT3 is constitutively expressed, and under physiological conditions its expression is tightly controlled—also in myocardial cells (i.e., cardiomyocytes, endothelial cells, smooth muscle cells, and fibroblasts). STAT3 regulates the expression of genes encoding proteins mainly involved in angiogenesis, apoptosis, inflammation, and oxidative stress—the canonical function of STAT3 [6, 21, 36, 82, 117]. STATs canonical functions are too slow for acute protection [6, 59]. However, subacute cardioprotection by late IPC and late RIC (ischemic conditioning is induced 24 h before myocardial infarction) involves the canonical function of STAT3 [11, 113] and STAT5 [17]. STAT3 activation during late preconditioning upregulates the expression of anti-apoptotic and cytoprotective proteins (e.g., cyclooxygenase 2, heme oxygenase-1, manganese sodium dismutase, myeloid leukemia protein 1, apoptosis regulator protein Bcl-2 family, c-FLIP a natural homologue of caspase 8, heat shock protein 70) [11, 17, 113] (Fig. 1). In contrast to the acute non-canonical and the subacute canonical STAT3 activation which serve a protective function, chronic STAT3 activation after myocardial infarction contributes to inflammatory processes and cardiac remodeling [6, 33, 34, 36, 55]. Such chronic STAT3 activation occurs mostly in macrophages which invade from the circulating blood and are recruited from bone marrow and spleen [112]. Up to four days after myocardial infarction, macrophages initiate or maintain inflammatory processes that contribute to debris clearance. Later on, however, macrophages express anti-inflammatory cytokines which then stimulate scar formation and angiogenesis (Fig. 1). The influence of chronic STAT3 activation after myocardial infarction on the processes described above is based on studies in rodents in the absence of a cardioprotective intervention, for further details please see: [6, 33, 34, 36, 55]. Importantly, chronic systemic STAT3 activation may also promote malignant transformation, which is of concern because RIC is a systemic phenomenon that may through STAT3 activation also promote cancer [46, 53].

Outside the nucleus, the non-canonical function of STAT3 plays a unique role in acute cardioprotection: in contrast to all other cardioprotective signals, STAT3 activation is consistently not only associated with the reduction of infarct size by all ischemic conditioning procedures, but also causally involved in cardioprotection in all species tested to date [6, 43, 45, 67, 69]—also in in larger mammals, i.e., in pigs, which are more similar to humans in their cardiovascular physiology than rodents [54, 70]. In detail: Interleukin 6-like cytokines and TNF appear to be the major activators of the cardioprotective SAFE pathway [36]. Downstream of activation of sarcolemmal glycoprotein 130 or TNF receptors, the cytosolic SAFE pathway is activated [6, 43, 45, 67, 69]. In response to IPC [27, 30, 31], POCO [50], and RIC [88, 92, 98] in rodents [4, 27, 31, 88, 92, 98] and pigs [30, 50, 60, 63, 66, 71, 92, 93], STAT3 is phosphorylated at tyr705. The causal involvement of STAT3 activation in infarct size reduction by different types of ischemic conditioning was demonstrated by pharmacological blockade of STAT3 activation [6, 43, 45, 67, 69]. In STAT3 knock-out mice and aged mice (which had reduced STAT3 protein), a more potent local ischemic conditioning stimulus overcame the STAT3-associated loss of cardioprotection [5], suggesting that other cardioprotective pathways are also involved. Apart from and in addition to a potential activation of NO/PKG and RISK pathways, STAT5 may have been activated. STAT5 knock-out in mice prevented infarct size reduction by local ischemic conditioning [114]. The ser727 phosphorylation of STAT3 was described in crosstalk with key proteins of the RISK pathway [36]. Present data suggest a dynamic balance between STAT3 phosphorylation at tyr705 and ser727 for STAT3 transcriptional activity; whether this may also play a role in cardioprotection is unclear. Notably, only STAT3/5 activation induced by ischemic conditioning appears to be causal for infarct size reduction, whereas basal STAT3/5 activity is not relevant for infarct size—neither pharmacological blockade nor knock-out increases infarct size per se.

The common intracellular target of all cardioprotective pathways, including the SAFE pathway, are mitochondria [8]. As such, mitochondria are critical elements of cardiomyocyte function and viability [12, 25, 32, 78, 91], and preservation of mitochondrial function is central for the reduction of ischemia/reperfusion injury [8, 43, 45] (Fig. 1). There is a prior comprehensive review on the non-canonical function of STAT3 in cardioprotection by targeting mitochondrial function [21]. The focus of my present somewhat personal and opinionated article is on the state-of-the-art of mitochondrial STAT3 and its potential causal role in cardioprotective strategies (Table 1). Mitochondrial STAT3 has been detected across species, in myocardium from mice [7, 85, 86, 96], rats [7, 84, 99, 109, 110], and pigs [50, 85].

**Table 1:** Studies on mitochondrial STAT3 and its function in myocardial tissue

Author, journal, year	Species	Import	Localization	Phosphorylation site	Function of mitochondrial STAT3
Wegrzyn et al., Science, 2009 [107]	Mouse, Pro-B cells wild-type vs. STAT3 knock-out	GRIM-19	Inner mitochondrial membrane, matrix, complex I of ETC, interacting with GRIM-19	Serine727	Association between mitochondrial STAT3 deficiency and reduction of mitochondrial respiration
Boengler et al., Basic Res Cardiol, 2010 [7]	Mouse, wild-type vs. cardiomyocyte-specific STAT3 knock-out, rat	Tom20	Matrix	Serine727 Tyrosine705	Association between mitochondrial STAT3 deficiency (isolated from cardiomyocyte-specific STAT3 knock-out) or inhibition of mitochondrial STAT3 activation (via stattic in wild-type mitochondria) and reduction of mitochondrial respiration and MPTP opening
Phillips et al., J Biol Chem, 2010 [85]	Mouse, Pig	Evidence for mitochondrial STAT3 without studies on import localization and phosphorylation			Questionable function of mitochondrial STAT3, ratio of ETC complex proteins to STAT3 is ~ 10^5^
Szczepanek et al., J Biol Chem, 2011 [96]	Mouse, wild-type vs. cardiomyocyte overexpression of a DNA-binding mutant of STAT3 containing a mitochondrial target sequence	Evidence for mitochondrial STAT3 without studies on import localization and phosphorylation			Protection against ischemic damage on complex I and decreased ROS formation from complex I during ischemia via mitochondrial STAT3 overexpression
Heusch et al., Circ Res, 2011 [50]	Pig	–	–	Tyrosine705	Causal involvement of activated mitochondrial STAT3 with POCO in preservation of complex I respiration and MPTP opening (proven via JAK–STAT inhibition with AG 490 application in vivo and inhibition of mitochondrial STAT3 activation via stattic in isolated mitochondria)
Qiu et al., Circulation, 2011 [86]	Mouse, wild-type vs. HSP22 knock-out	HSP22	Inner mitochondrial membrane, matrix	Serine727	Association of HSP22 deficiency with reduction of mitochondrial STAT3 and reduction of mitochondrial respiration
Tammineni et al., J Biol Chem, 2013 [99]	Rat	GRIM-19	Inner mitochondrial membrane, complex I of ETC, interacting with GRIM-19	Serine727	Relevance of STAT3 serine727 phosphorylation for GRIM-19-mediated integration of STAT3 into mitochondrial complex I
Boengler et al., Curr Pharm Des, 2013 [10]	Rat	No assessment of the mitochondrial STAT3 protein, only functional studies			Association of reduced mitochondrial STAT3 activation (via stattic in isolated mitochondria) with a reduction of mitochondrial respiration, ATP production, MPTP opening and increased ROS formation
Penna et al., Basic Res Cardiol, 2013 [84]	Rat	–	–	Serine727 Tyrosine705	Activated mitochondrial STAT3 with IPC but not with POCO
Wu et al., J Mol Cell Cardiol, 2015 [110]	Rat	–	–	Serine727	Activated mitochondrial STAT3 with pIHH; association of reduced mitochondrial STAT3 activation (via AG490) with a reduction of MPTP opening during reperfusion
Wu et al., Basic Res Cardiol, 2019 [109]	Rat	–	Inner mitochondrial membrane, colocalization/interaction with the N-terminal domain of the mitochondrial calcium uniporter	Serine727	Activated mitochondrial STAT3 with poIHH; association with a reduction in calcium overload during reperfusion
Harhous et al., J Mol Cell Cardiol, 2019 [35]	Mouse	No evidence for mitochondrial STAT3 in pure mitochondria under basal conditions and after hypoxia/reoxygenation			No evidence for functionally relevant mitochondrial STAT3

AG490, tyrphostin B42, a JAK–STAT inhibitor; *ATP* adenosine triphosphate, *ETC* electron transport chain, *GRIM-19* gene associated with retinoid interferon-induced cell mortality 19, *HSP22* heat shock protein 22, *IPC* ischemic preconditioning, *JAK* Janus kinase, *MPTP* mitochondrial permeability transition pore, *Tom20* mitochondrial import receptor subunit Tom20, *pIHH* preconditioning via intermittent hypobaric hypoxia, *POCO* ischemic postconditioning, *poIHH* postconditioning via intermittent hypobaric hypoxia, *ROS* reactive oxygen species, *STAT3* signal transducer and activator of transcription 3, *stattic* STAT3 inhibitory compound 6 nitrobenzol(b)thiopene 1,1-dioxide

## STAT3 phosphorylation site, import into, and localization within mitochondria

**Fig. 2:**
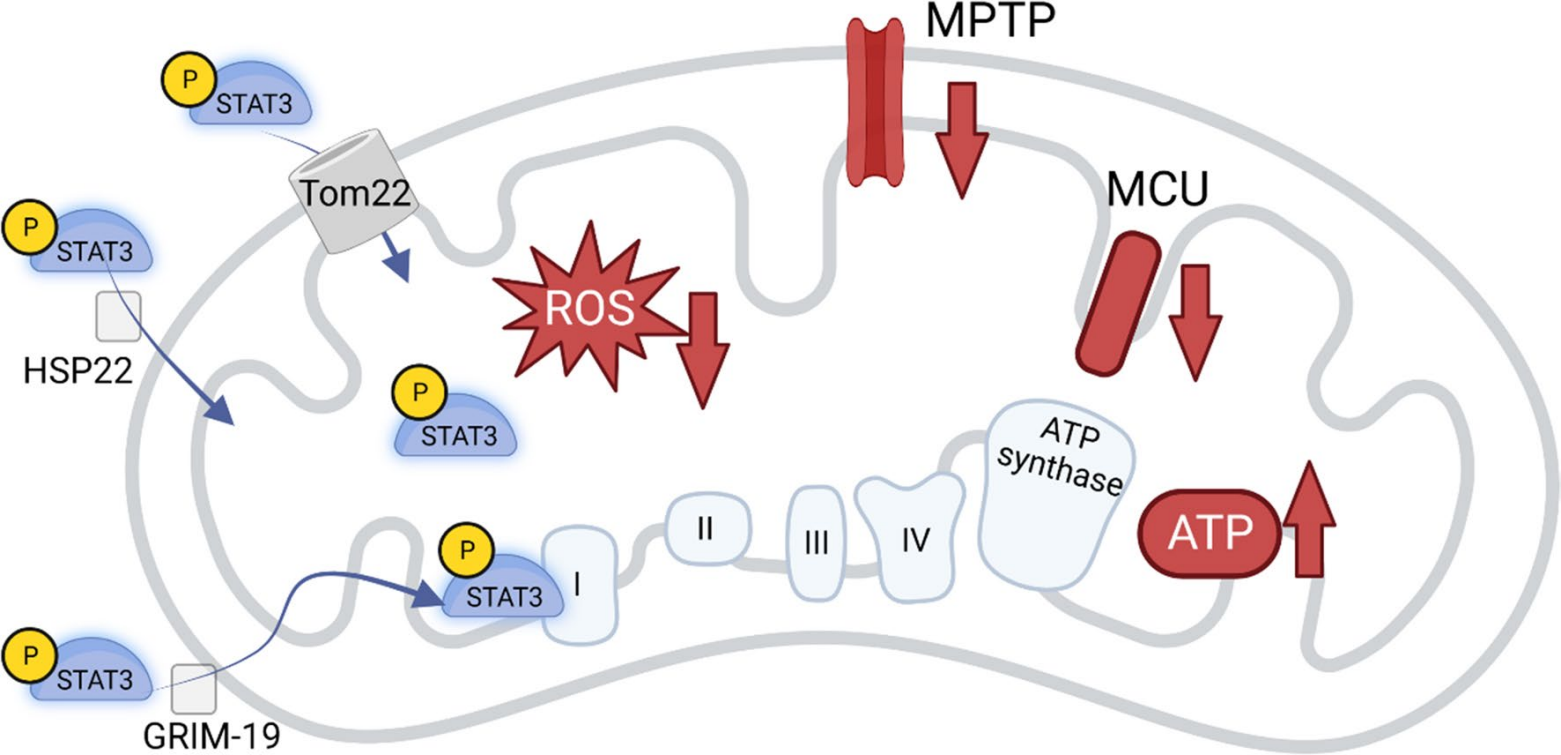
Schematic overview of STAT3 import, localization, and function in mitochondria; created with BioRender.com. I, II, II, IV indicates respiratory chain complexes; *ATP* adenosine triphosphate, *GRIM-19* gene associated with retinoid interferon-induced cell mortality 19, *HSP22* heat shock protein 22, *MCU* mitochondrial calcium uniporter, *MPTP* mitochondrial permeability transition pore, *Tom20* mitochondrial import receptor subunit Tom20, *ROS* reactive oxygen species, *STAT3* signal transducer and activator of transcription 3

In vitro studies with isolated rat heart mitochondria and labeled STAT3 proposed an energy-dependent import of STAT3 mediated through chaperone-like activity of a complex I subunit protein—the protein of the gene associated with retinoid interferon-induced cell mortality 19 (GRIM-19, Fig. 2) [99]. In rat left ventricular protein extracts, mitochondrial import receptor subunit Tom20 co-immunoprecipitated with ser727 STAT3 and total STAT3 [7], suggesting a Tom20-dependent import. In heat shock protein 22 (HSP22) knock-out mice, STAT3 translocation into the mitochondria was reduced, and HSP22 co-immunoprecipitated with total STAT3 [86], indicating another import mechanism (Fig. 2). The initial study suggested that ser727 phosphorylation is required for STAT translocation to mitochondria, a ser727 mutation in STAT3 reduced the import and the STAT3 GRIM-19 assembly in isolated rat heart mitochondria [99]. As mentioned above, it is still unclear whether ser727 phosphorylation of STAT3 is indeed causally involved in infarct size reduction. However, while in mouse [86, 107] and rat [84, 109, 110] heart mitochondria ser727 phosphorylation was described, a tyr705 phosphorylation has been detected in rat heart mitochondria in addition to the ser727 phosphorylation [7, 84], and in pig heart mitochondria, only the tyr705 phosphorylation was reported [50]. Beside potential species-specific differences, it is also conceivable that simple methodological reasons, such as the antibodies used and their species-specificity, may have led to the reported differences. Regardless of the exact phosphorylation site, given that phosphorylation of STAT3 is a prerequisite for mitochondrial import, the cytosolic non-canonical function and, thus, phosphorylation of STAT3 seems to be a prerequisite for mitochondrial import of STAT3.

Using enzymatic digestion of isolated mouse and rat heart mitochondria and Western blot technique with specific marker proteins for intra-mitochondrial localization, STAT3 was identified in the matrix of mitochondria [7, 86, 107] as well as in the inner mitochondrial membrane [86, 99]. Using immunofluorescence imaging, a colocalization of STAT3 with the mitochondrial calcium uniporter—which is localized in the inner mitochondrial membrane—was identified in rat hearts. Co-immunoprecipitation confirmed an interaction with the N-terminal domain of the mitochondrial calcium uniporter and STAT3 in isolated rat cardiomyocytes [109] (Fig. 2, Table 1).

## Function of mitochondrial STAT3

Phillips et al. had questioned the functional relevance of mitochondrial STAT3 by quantitative biochemical analyses: the abundance of mitochondrial STAT3 compared with respiratory chain proteins was low, with a ratio of electron transport complex proteins to STAT3 of ~ 10^5^, and relevance to adenosine triphosphate (ATP) production was, thus, ruled out [85]. However, studies on mitochondrial function indicate the opposite. Cardiomyocyte-specific STAT3 knock-out mice had not only less mitochondrial STAT3, but also selective defects in complex I of the electron transport chain [7]. Cardiomyocyte-specific overexpression of mitochondrial STAT3 in mice improved complex I respiration during ischemia [96] (Fig. 1). In mice that overexpress a mitochondrially targeted, transcriptionally inactive STAT3 in cardiomyocytes, a partial and persistent blockade of complex I was evident. Smaller infarct size in these mice in comparison to wild-type mice was associated with an attenuated reactive oxygen species release and attenuation of mitochondrial permeability transition pore opening at the onset of reperfusion [97]. In isolated rat heart mitochondria, pharmacological STAT3 inhibition with stattic (STAT3 inhibitory compound 6 nitrobenzol(b)thiopene 1,1-dioxide) reduced mitochondrial respiration and ATP production; opening of the mitochondrial permeability transition pore and generation of reactive oxygen species were reduced [10] (Fig. 2). There is evidence for STAT3 not only in isolated mitochondria from rodent hearts but also in those from larger mammals. In mitochondria isolated from pig myocardium, POCO induced an increase in mitochondrial STAT3 phosphorylation. This phosphorylation improved mitochondrial complex I respiration and increased calcium retention capacity [50]. The biochemical mechanisms, however, by which mitochondrial STAT3 regulates the electron transport chain complex activities and mitochondrial permeability transition pore opening are not clear. GRIM-19 enhanced in isolated rat heart mitochondria the integration of STAT3 into complex I [99], indicating a direct interaction of STAT3 with complex I activity (Fig. 2, Table 1). In a postconditioning protocol by intermittent hypobaric hypoxia, the colocalization of STAT3 and the mitochondrial calcium uniporter was associated with a reduction in mitochondrial calcium overload during reperfusion [109] (Fig. 2, Table 1).

## Causal evidence for mitochondrial STAT3 in cardioprotection

A number of studies have suggested an association between mitochondrial STAT3 activation and infarct size reduction during maneuvers such as IPC [84] or intermittent hyperbaric hypoxia [109, 110]. However, there is only one study describing a causal evidence for mitochondrial STAT3 in infarct size reduction with POCO. In pigs, improved mitochondrial function after POCO was causally related to increased STAT3 tyr705 phosphorylation, but not to total STAT3. AG490 (tyrphostin B42), a JAK–STAT inhibitor, when administered in vivo before POCO, not only abolished the reduction in infarct size but also the protective effect on mitochondrial function. In vitro, stattic (a non-peptide small molecule inhibitor that inhibits STAT3 activity by binding to its SH2 region, which is essential for the tyr705 phosphorylation) abrogated better preservation of mitochondrial function when isolated after POCO. The fact that in mitochondria only STAT3 tyr705 was increased, but not total STAT3, suggests that STAT3 import into mitochondria does not play a role in acute cardioprotection [50]. In rats, POCO did not activate mitochondrial STAT3, but activated proteins of the RISK pathway which were subsequently translocated to the mitochondria [84]. In this study, however, it remained unclear whether or not the POCO maneuver that was performed actually reduced the infarct size (Table 1).

## Critical considerations on the detection and function of mitochondrial STAT3

For methodological reasons, most studies investigated mitochondria isolated from the total myocardium; thus, mitochondrial fractions from all myocardial cell types are included. The estimated proportion of cardiomyocytes in the left ventricular myocardium in rodents is between 75% (estimated from cell volume data) and 50% (estimated from nuclei data) [73, 102]. In adult human ventricular myocardium, transcriptome analysis identified 49% cardiomyocytes, whereas studies using nuclear labeling techniques found only about 30% cardiomyocytes [74]. Since mitochondria in cardiomyocytes occupy about 30–40% of the total volume [75], and mitochondrial density in other cells (e.g., endothelial cells) is much lower at 2–5% [58], studies on mitochondria isolated from myocardial tissue mainly refer to cardiomyocyte mitochondria, but not exclusively. Considering that STAT3 activation also plays an important role in non-cardiomyocyte cells such as fibroblasts and endothelial cells, modulating there cell proliferation, differentiation, oxidative stress, cell metabolism, and survival [33], it is reasonable to assume that mitochondrial STAT3 may originate not exclusively from cardiomyocytes. Of note, the above numerical estimates may vary species-specifically, as there are significant species-specific differences in the cellular composition between mouse, rat, and human hearts [3]. In cardiomyocytes, it has been estimated that the majority of mitochondria in cardiomyocytes are interfibrillar mitochondria (IFM), a much smaller proportion are subsarcolemmal mitochondria (SSM), and the smallest proportion are perinuclear mitochondria [87]. Although many studies in rodents have documented differences between SSM and IFM not only in location, but also in function (e.g.. see [9, 56, 68, 83]), a functional difference in ischemic reperfused myocardium, however, was not confirmed in a study in pigs [16]. In rat myocardium, STAT3 was quantified in preparations of SSM and IFM [7]. Because selective isolation of perinuclear mitochondria is methodologically difficult [68], perinuclear mitochondria have not been studied in this regard.

Based on the findings discussed above, it is reasonable to assume that mitochondrial STAT3 plays a critical role in cardioprotection (Table 1). Again, however, using 3 different proteomic approaches in mouse myocardium, the abundance of mitochondrial STAT3 was estimated to be very low (10% of the total cytoplasmic STAT3 and a ratio of electron transport complex proteins to STAT3 of ~ 10^5^) [85], so a relevance of protein–protein interaction in the mitochondria seems indeed questionable. The low abundance of STAT3 in mitochondria is indirectly confirmed by the fact that without prior immunoprecipitation of STAT3, e.g., as done in the study by Boengler et al. [7], detection in isolated mouse mitochondria failed [97]. Further, in a Percoll-purified mitochondrial preparation from mouse myocardium, STAT3 was neither detectable under baseline conditions nor after hypoxia/reoxygenation via Western blot, and confocal imaging showed no colocalization of STAT3 signal with mitochondrial proteins. In this study, only a STAT3 overexpression in a H9C2 cardiomyoblast cell line led to detectable translocation of STAT3 into mitochondria [35]. Overall, it seems most plausible that differences in mitochondrial STAT3 detection are due to the methods used (different purification, enrichment, and denaturation protocols, antibodies, etc.) and which of the available results best reflects the biological reality remains unclear. The functional studies in genetically modified mice and on mitochondria with pharmacological blockade are definitely not affected by these methodological aspects and clearly indicate a relevance of mitochondrial STAT3 for mitochondrial function. However, both the use of genetically modified animal models and the use of pharmacological blockers have different, but also fundamental limitations (e.g., for animal models, the genetic compensation of the knock-out and for pharmacological blockers side effects, non-specificity, toxicity, etc.).

In conclusion, the precise function and importance of mitochondrial STAT3 during cardioprotection is still unclear. Finally, to what extent the acute cardioprotective effect is mediated by cytosolic STAT3 or mitochondrial STAT3 activation remains open.

## Evidence for STAT3/5 in humans

Indeed, there is even evidence from the human myocardium that STAT is associated with cardioprotection by ischemic conditioning. In left ventricular biopsies, taken at early reperfusion after cardioplegic ischemic arrest from patients undergoing bypass surgery [51], the activation and expression of 22 signaling proteins, key signaling proteins of the NO/PKG, RISK and SAFE pathway were analyzed using Western blot analysis. Among these 22 proteins, only the activation of STAT5 was associated with reduction of perioperative myocardial injury by RIC [51]. Confirming results in right ventricular outflow tract biopsies of children undergoing tetralogy of Fallot repair surgery, activation of STAT3 and STAT5 was also associated with perioperative myocardial protection by RIC [111]—highlighting again the potentially relevant role of STATs in cardioprotection. Even when human myocardium is investigated, cardioprotective strategies improve mitochondrial function [1, 62, 111]. Since mitochondrial STAT has not yet been detected in human myocardium, it is difficult to predict the relevance of the available data for the translation to patients. In principle, also other members of the STAT family (STAT1, STAT2, STAT5, and STAT6) are present in the mitochondria [7, 76]. Because there are several independent lines of evidence that STAT is associated with cardioprotection in human myocardium, further and more detailed analysis of (mitochondrial) STAT signaling in human myocardium is warranted.

## Lack of STAT3 responsiveness: a novel confounding factor

Ossabaw minipigs, a particular strain of minipigs, are characterized by a genotype associated with a thrifty phenotype. Like humans, they develop a metabolic syndrome when fed a hypercaloric, atherogenic diet and consequently coronary atherosclerosis and occasional myocardial infarction [95, 101, 118]. The unequivocally strongest and most robust stimulus for cardioprotection, IPC, failed to reduce infarct size in a power analysis-based experimental design in these Ossabaw minipigs—even when they were lean and only predisposed to metabolic syndrome. Bioinformatic analysis of genetic differences between these Ossabaw minipigs and Göttingen minipigs, in which IPC confers robust protection, identified several clusters of protein-coding genes. One cluster was related to mitochondrial and one to JAK–STAT signaling. Indeed, the lack of infarct size reduction with IPC in the Ossabaw minipigs was associated with a lack of STAT3 activation in the myocardium [64]. RIC also failed to reduce infarct size in the Ossabaw minipigs, but RIC still induced a release of cardioprotective factors into the circulation in these Ossabaw minipigs, as evidenced by their protective effect after transfer to isolated rat hearts; thus, the lack of cardioprotection was attributed to myocardial—i.e., STAT3-dependent—non-responsiveness [72]. These studies in Ossabaw minipigs once again independently underscored the importance of STAT3 signaling for cardioprotection. However, the extent to which mitochondrial STAT3 plays here a role is unclear.

The neutral results of this prospectively designed experimental study in the Ossabaw minipigs are similar to the neutral results of several larger all-comer randomized controlled trials on RIC in patients undergoing interventional reperfusion of myocardial infarction [39] or cardiovascular surgery [37, 77]. In addition to the often discussed confounders such as comorbidities and co-medications which are typical for patients with acute myocardial infarction [61], genetic variance may be newly considered as a potential confounder for cardioprotective measures [48, 104]. In this context, it is noteworthy that STAT3 levels were reduced in aged mice and that this reduction in STAT3 levels was associated with a loss of the cardioprotective effect of POCO [5], suggesting that in addition to a genetic heterogeneity also age may act as confounding factor. A genetic heterogeneity of STATs exists also in humans. The European Lymphoma Risk Study identified human single-nucleotide polymorphisms belonging to the JAK–STAT pathway—including STAT3 and STAT5 [14]. The unique Ossabaw minipig strain may, therefore, be a suitable model to further investigate a genetically determined lack of susceptibility to cardioprotection and to develop therapeutic strategies and to possibly circumvent this blockade of cardioprotective signaling.

## Cardioprotective effects of SGLT2 inhibitors through STAT3 activation

Sodium–glucose cotransporter 2 (SGLT2) inhibitors—also known as gliflozins—are a class of drugs originally developed to treat type 2 diabetes via inhibition of the sodium–glucose transport protein 2 in the kidney [2]. However, clinical trials have impressively shown that in addition to lowering blood glucose levels, gliflozins also significantly improve cardiovascular outcomes in patients with and without type 2 diabetes, indicating multifaceted cardioprotective effects that are beyond inhibition of the sodium–glucose transport protein 2 in the kidneys [2]. The larger clinical trials did not identify a reduction in the incidence of acute coronary syndromes, however, dapagliflozin and empagliflozin reduced the incidence of recurrent myocardial infarction, possibly reflecting attenuated ischemia/reperfusion injury [2]. Indeed, a recent study in SGLT2 knock-out mice demonstrated that the infarct size reduction by empagliflozin was completely independent of its initial target, sodium–glucose transport protein 2 inhibition [18]. Among all potential mediators discussed in the context of gliflozin-induced but SGLT2-independent cardioprotection [2], myocardial STAT3 activation could be a common denominator. In fact, one-week of treatment with dapagliflozin and empagliflozin before induction of myocardial infarction in mice reduced infarct size, and STAT3 activation was causally involved. In association with cardioprotection by empaliflozin, mitochondrial complex I and II respiration was preserved after myocardial infarction [81]. A more acute administration of empaliflozin (4 or 24 h before myocardial infarction in mice), however, failed to reduce myocardial infarct size, and STAT3 was not activated [80]. However, 24 h pretreatment of empaliflozin increased STAT3-dependently the survival of cultured human endothelial cells after hypoxia/reoxygenation [80]. Coronary endothelial cells are relative resistant to ischemia, but with endothelial swelling obstructing capillary blood flow, they contribute to microvascular damage in response to myocardial ischemia/reperfusion injury, and microvascular obstruction has a strong impact on patient prognosis [38]. Notably, knock-out of endothelial STAT3 in mice resulted in reduced recovery of left ventricular function during reperfusion after myocardial infarction [106]. In the EMMY trial [105] in patients with recent myocardial infarction, daily administration of SGLT2 inhibitors (with onset no later than 72 h after interventional reperfusion) preserved left ventricular function, possibly through anti-inflammatory effects [28]. However, a number of issues on the role of STAT3 in the gliflozin action remain to be resolved: Is, in response to gliflozins, STAT also activated in the human heart? In cardiomyocytes or endothelial cells, possibly also in inflammatory cells? Does STAT3 serve a canonical or non-canonical function? Are mitochondria involved?

Recently, also other compounds have been described that activate STAT3 and are causally involved in cardioprotection under experimental conditions. The anabolic steroid nandrolone decanoate [22] and dexmedetomidine—a selective alpha 2 adrenoceptor agonist [19] reduced infarct size/biomarker release reflecting myocardial injury in rodents via STAT3 activation. Again, improved mitochondrial function was also associated with cardioprotection. Thus, these agents may also be of interest for use in patients. For these agents, it is even more important to conduct further studies to determine the extent to which the results of the initial experimental studies can be transferred to patients.

## Conclusion

STAT3 plays an important role in cardioprotection, also in the human heart. In animal models, there is evidence of mitochondrial STAT3 involved in mitochondrial function. The assumed causal relationship between mitochondrial STAT3 and cardioprotection is based on only one study in pigs. Data from human myocardium on mitochondrial STAT are lacking. There is a primordial non-responsiveness to cardioprotection in pigs which involves STAT3. Combination therapies that acutely activate STAT3—possibly mitochondrial STAT3—through different pathways could therefore be particularly effective, as they could bypass possible blockades upstream of STAT3. The timing of STAT activation seems to be relevant for therapeutic approaches. Acute activation and the non-canonical function of STAT3 are cardioprotective, subacute activation of canonical STAT3 function in late preconditioning is also cardioprotective. However, chronic activation of canonical STAT3 function may be more detrimental than beneficial.
